# Evaluation of Enhanced Attention to Local Detail in Anorexia Nervosa Using the Embedded Figures Test; an fMRI Study

**DOI:** 10.1371/journal.pone.0063964

**Published:** 2013-05-14

**Authors:** Leon Fonville, Nick P. Lao-Kaim, Vincent Giampietro, Frederique Van den Eynde, Helen Davies, Naima Lounes, Christopher Andrew, Jeffrey Dalton, Andrew Simmons, Steven C.R. Williams, Simon Baron-Cohen, Kate Tchanturia

**Affiliations:** 1 King’s College London, Institute of Psychiatry, Department of Psychological Medicine, London, United Kingdom; 2 King’s College London, Institute of Psychiatry, Department of Neuroimaging, London, United Kingdom; 3 NIHR Biomedical Research Centre for Mental Health at South London and Maudsley NHS Foundation Trust and Institute of Psychiatry, King’s College, London, United Kingdom; 4 Autism Research Centre, University of Cambridge, Department of Psychiatry, Cambridge, United Kingdom; 5 Eating Disorders Program, Douglas University Institute, Psychiatry Department, McGill University, Montreal, Quebec, Canada; University of Granada, Spain

## Abstract

The behavioural literature in anorexia nervosa and autism spectrum disorders has indicated an overlap in cognitive profiles. One such domain is the enhancement of local processing over global processing. While functional imaging studies of autism spectrum disorder have revealed differential neural patterns compared to controls in response to tests of local versus global processing, no studies have explored such effects in anorexia nervosa. This study uses functional magnetic resonance imaging in conjunction with the embedded figures test, to explore the neural correlates of this enhanced attention to detail in the largest anorexia nervosa cohort to date. On the embedded figures tests participants are required to indicate which of two complex figures contains a simple geometrical shape. The findings indicate that whilst healthy controls showed greater accuracy on the task than people with anorexia nervosa, different brain regions were recruited. Healthy controls showed greater activation in the precuneus whilst people with anorexia nervosa showed greater activation in the fusiform gyrus. This suggests that different cognitive strategies were used to perform the task, i.e. healthy controls demonstrated greater emphasis on visuospatial searching and people with anorexia nervosa employed a more object recognition-based approach. This is in accordance with previous findings in autism spectrum disorder using a similar methodology and has implications for therapies addressing the appropriate adjustment of cognitive strategies in anorexia nervosa.

## Introduction

Anorexia nervosa (AN) is an eating disorder primarily affecting young women and is associated with the highest mortality rates amongst all eating disorders [1]. It is characterized by a refusal to maintain normal body weight, an intense fear of gaining weight despite being underweight, a disturbance in the perception of one’s own body weight or shape and, in postmenarcheal women, an absence of at least three consecutive menstrual cycles. AN is further divided into a restricting subtype (AN-R) and a binge-eating/purging subtype (AN-BP), the latter being defined by the additional criteria of regular binge-eating or purging behaviour, which may include vomiting and the use of diuretics, laxatives and enemas [2].

It has been suggested that part of these maladaptive behaviours are associated with neurocognition [3], relating to a profile of inefficient set-shifting, cognitive flexibility, central coherence, attention, social cognition, and long-term and visuospatial memory [4]–[9]. Similarities between the cognitive profile of AN and that of Autism Spectrum Disorders (ASD) have been suggested [10], [11] and it has been noted that ASD is overrepresented in AN populations [12]–[15]. A recent study directly comparing ASD and AN performance on tasks measuring empathy, executive function and central coherence found considerable overlap in cognitive profiles [16]. In particular, studies in AN have reported a tendency to focus on local detail at the expense of global processing (Table S1) [17]–[21], also defined as weak central coherence in the ASD literature [22]. However, many of the assumptions for weak central coherence are untested [23], therefore we adopt the term *enhanced attention to local detail*. Similar to ASD, people with AN exhibit faster reaction times and greater accuracy on paradigms such as the Embedded Figures Test (EFT) [24] that require greater attention to detail over global processing [18], [21], [25].

Previous studies have attempted to identify the neural correlates of weak central coherence or enhanced attention to local detail in ASD using the EFT with functional magnetic resonance imaging (fMRI), but have been limited by small sample sizes and inconsistent methodologies, making comparisons difficult (Table S2). Those employing a low-level baseline have reported that, compared to healthy controls (HC), people with ASD show greater activation in occipital and parietal regions but less activation in frontal regions [26], [27]. A pilot investigation in the parents of children with ASD found less activation in occipital regions compared to age-matched HC, regardless of gender [28]. Conversely, the analysis of the HC group showed a gender effect in which females showed greater activation than males in these regions. Studies using a “matching figures task” similar to the EFT as a baseline, in an attempt to isolate the visuospatial searching component, have found either no difference [29] or reported less activation in occipital regions and greater activation in temporal regions in ASD when compared to HC [30]. The same group also found greater task-related deactivation of parietal regions and the posterior cingulate cortex, areas corresponding to the default mode network, in HC as compared to ASD as well as their unaffected siblings [31].

With regards to AN, a recent study found poorer visual memory and weaker central coherence on the Rey-Osterrieth Complex Figure Test (RCFT) [32], as well as decreased functional connectivity using resting-state fMRI in ventral occipital regions and the somatosensory cortex in both current and weight-recovered AN [33]. However, it is still unclear whether the neurobiology underlying behavioural performance on tasks assessing enhanced attention to local detail is similar in ASD and AN. Thus, the current fMRI study aims to examine the neural correlates of enhanced attention to local detail in AN using the EFT.

We hypothesize that people with AN will show superior performance, defined as faster reaction times and greater accuracy, alongside greater activation in occipital and parietal regions as compared to HC.

## Methods

### Participants

A total of seventy-two participants took part in this study. Thirty-five individuals with a current diagnosis of AN were recruited from the hospital and community services of the South London and Maudsley (SLaM) National Health Service Trust and from an online advertisement on the b-eat website (beating eating disorders – http://www.b-eat.co.uk), the UK’s largest eating disorder charity. Twenty-eight (80%) were diagnosed as restrictive (AN-R) and seven (20%) as binge-purging (AN-BP). Sixteen (46%) reported taking antidepressant or anti-anxiety medication. Thirty-seven age-matched healthy individuals with no personal or family history of eating disorders were recruited from the community, staff and students of the Institute of Psychiatry, King’s College London. Two healthy participants were excluded from further analysis due to currently taking antidepressant medication. Body mass index (BMI), medication, age of onset and duration of illness were obtained on the day of testing. The research version of the structured clinical interview for DSM disorders (SCID) was administered by a trained researcher to assess Axis I psychiatric comorbidities in the patients with AN and was used as a screening tool for the healthy controls. The National Adult Reading Test (NART) was used to estimate IQ [34]. Participants consent was obtained according to the Declaration of Helsinki (BMJ 1991; 302∶1194) and was approved by the National Research Ethics Committee London Bentham (11-LO-0952).

### Clinical Measures

All participants completed self-report questionnaires prior to the scanning procedure to assess levels of anxiety, depression, eating disorder-related behaviour and obsessive compulsive symptoms. The Hospital Anxiety and Depression Scale (HADS) is a measure consisting of 14 items to assess overall severity of depression and anxiety (clinical threshold total = 10) [35]. The Eating Disorders Examination Questionnaire (EDE-Q) consists of 36 items that evaluate a participant’s eating behaviour over the past 28 days [36]. The Obsessive-Compulsive Inventory Revised (OCI-R) is an 18-item list of first person statements which a participant has to rate according to the level of distress they felt when they experienced those statements over the past month [37].

### Embedded Figures Test

**Figure 1:**
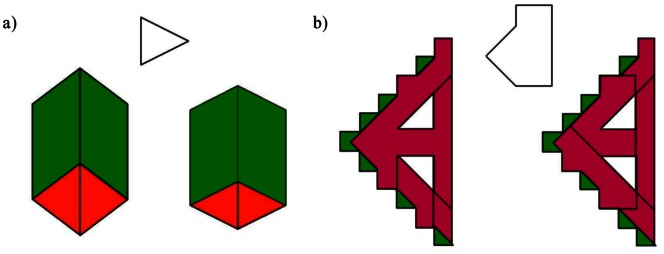
Example trials of the Embedded Figures Test as they appeared on the projection screen. a) Simple embedded figure trial (SEF) b) Complex embedded figure trial (CEF).

Each trial of the EFT consisted of the simultaneous presentation of a target geometrical shape and two more complicated figures. The stimuli were projected onto a screen and viewed using a mirror mounted on the headcoil. Participants were required to indicate, using a joystick (left or right), which of the two figures contained the target geometrical shape. The level of difficulty was manipulated by displaying figures that were either simple (SEF) or complex (CEF) in their construction (Figure 1).

A total of 18 SEF and 18 CEF unique trials, each lasting for 10 seconds, were presented in alternating blocks of 3 trials. Once the participant responded, their choice was indicated by highlighting the selection on screen. Performance (correct or incorrect) and response times (latency between stimulus onset and participant response) were recorded and trials in which the full 10 seconds elapsed without a response were taken as incorrect. A low-level baseline (LLB) consisting of a black screen with a white fixation cross was presented at the beginning, in the middle and at the end of the experimental run, for 30 seconds each, for which participants were instructed to focus on the cross. The total duration of the task was 450 seconds and all participants were given training sessions to ensure that they understood the task.

### Image Acquisition

Magnetic resonance imaging was performed using a 1.5T GE Signa HDx TwinSpeed MRI scanner (GE-Medical Systems, Wisconsin) at the Centre for Neuroimaging Sciences, Institute of Psychiatry, King’s College London. The body coil was used for radio frequency (RF) transmission, with an 8 channel head coil for RF reception. To facilitate normalisation to standard space, a high resolution gradient echo EPI was acquired at 43 near-axial slices parallel to the anterior commissure-posterior commissure (AC-PC) line with the following parameters: repetition time (TR) = 3000 ms, echo time (TE) = 40 ms, flip angle = 90°, slice thickness = 3 mm, inter-slice gap = 0.3 mm, field of view (FoV) = 240×240 mm, matrix size = 128×128 and in-plane voxel size of 1.88×1.88 mm.

T2*-weighted gradient echo EPI images depicting blood-oxygen-level-dependent (BOLD) contrasts were acquired at 25 near-axial slices parallel to the AC-PC line (TR = 2000 ms, TE = 40 ms, flip angle = 70°, slice thickness = 5 mm, inter-slice gap = 0.5 mm, FoV = 240×240 mm, matrix size = 64×64, in-plane voxel size = 3.75×3.75 mm). A total of 225 T2*-weighted whole brain volumes were acquired for each subject. Data quality was assured using an automated quality control procedure [38].

### Demographic, Clinical and Performance Analysis

Demographic, clinical and performance data were analysed using SPSS 20 [39]. An assessment of normality was performed for all data using the Shapiro-Wilks test. When data were normally distributed, the Student t-test was used to examine between-group differences. When normality was violated, the non-parametric Mann-Whitney U test was used. With regards to the assessment of correlations within the data, Pearson’s *r* was used when the data were normally distributed and Spearman’s rho (ρ) when data were not normally distributed.

### Imaging Analysis

Imaging data was analysed using XBAM version 4.1 (http://brainmap.it), an fMRI software package developed at King’s College London, Institute of Psychiatry [40]. Data were first processed to minimize motion related artefacts [41]. Following realignment, images were smoothed using an 8.83 mm full-width half-maximum Gaussian filter.

Responses to each condition were then detected by time-series analysis using a linear model in which each component of the experimental design was convolved separately with a pair of Poisson kernels (λ = 4 and 8 seconds) to allow variability in the haemodynamic delay. The best fit between the weighted sum of these convolutions and the time-series at each voxel was computed using the constrained BOLD effect model [42]. A goodness of fit statistic was then computed as the ratio of the sum of squares of deviations from the mean image intensity resulting from the model (over the whole time-series) to the sum of squares of deviations resulting from the residuals (SSQ ratio).

Following computation of the observed SSQ ratio at each voxel, the data were permuted by the wavelet-based method described in Bullmore et al [43]. The observed and permuted SSQ ratio maps for each individual were transformed into the standard space of Talairach and Tournoux [44] using a two-stage warping procedure [40]. Group maps of activated voxels were then computed using the median SSQ ratio at each voxel (over all individuals) in the observed and permuted data maps [40]. Computing intra and inter participant variations in effect separately constitutes a mixed effect approach, which is desirable in fMRI. Detection of activated regions was extended from voxel to 3D cluster-level using the method described by Bullmore et al [45].

Comparisons of responses between-groups at each condition and comparisons of responses between-conditions for each group separately were performed by fitting the data at each intracerebral voxel at which all participants have non-zero data using the linear model:
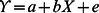
where ‘Y’ is the vector of SSQ ratios for each individual, ‘X’ is the contrast matrix for the inter-group/inter-condition contrast, ‘a’ is the mean effect across all individuals in the groups/conditions, ‘b’ is the computed group/condition difference and ‘e’ is a vector of residual errors. The model is fitted by minimising the sum of absolute deviations to reduce outlier effects. The null distribution of ‘b’ is computed by permuting data between-groups/conditions and refitting the above model a maximum of 50 times at each voxel and combining the data over all intracerebral voxels.

The interaction between group membership and task (SEF or CEF) was analysed using a split-plot analysis of variance (ANOVA) model in order to ascertain whether any brain regions exhibit group-dependent differences in within-group monotonic trends. Using the derived null distribution, all resulting 3D cluster-level maps were then thresholded in such a way as to yield less than one expected type I error cluster per map.

## Results

### Group Characteristics

Clinical and demographic data are summarized in Table 1. Patients and controls were of similar age, but there was a significant difference between groups in terms of IQ and BMI. Participants in the AN group showed higher levels of eating disorder symptomatology, depression, anxiety and obsessive compulsive symptoms.

**Table 1:** Clinical and demographic characteristics.

	AN	HC	Test Statistic	p
	N = 35	N = 35		
Age^a^	23 (9)	25 (4)	U = 492, z = −1.42	.156
Current BMI^b^	16.0 (1.6)	21.9 (1.9)	t(68) = 13.995	<.001
IQ^c^	110 (9)	117 (10)	U = 355, z = −2.733	.006
Years of Educationb d	15.8 (2.4)	17.9 (3.1)	t(66) = 3.292	.002
EDE-Q^c^	3.9 (1.7)	.4 (.6)	U = 1186, z = 7.094	<.001
HADS Depression^c^	10.5 (8.0)	0 (2)	U = 1171, z = 6.997	<.001
HADS Anxiety^c^	14.9 (3.8)	4.6 (3.2)	t(67) = −12.328	<.001
OCI-R^c^	22.5 (26.5)	4 (6)	U = 1107, z = 6.155	<.001
Duration of Illness^e^	8.9 (8.1)	–	–	–

a Data were normally distributed, therefore t-test statistics, mean and standard deviation are reported.

b Data were not normally distributed therefore Mann-Whitney *U* test statistics, mean and interquartile range are reported.

c Measures are based on thirty-four AN.

d Measures are based on thirty-three HC.

e Measures are based on thirty-one AN.

### EFT Performance

There were no differences in reaction time between the groups, however HC made significantly less errors on both the simple and complex trials (Table 2). No correlations were found in either group between performance (reaction time and accuracy) on the SEF or CEF and clinical and demographic measures.

**Table 2:** Reaction time and accuracy on simple figures (SEF) and complex figures (CEF).

	AN	HC	Test Statistic	p
	N = 35	N = 35		
SEF Reaction Time^b^	1.8 (1.2)	1.8 (.8)	U = 682, z = .816	.414
CEF Reaction Time^b^	5.7 (1.7)	5.6 (1.3)	U = 692, z = .934	.350
SEF Accuracy^b^	94.4 (11.1)	100 (5.6)	U = 453, z = −2.109	.035
CEF Accuracy^a^	64.4 (16.3)	72.1 (12.4)	t(68) = 2.203	.031

a Data were normally distributed therefore t-test statistics, mean and standard deviation are reported.

b Data were not normally distributed therefore Mann-Whitney *U* test statistics, mean and interquartile range are reported.

### Imaging Results

**Figure 2:**
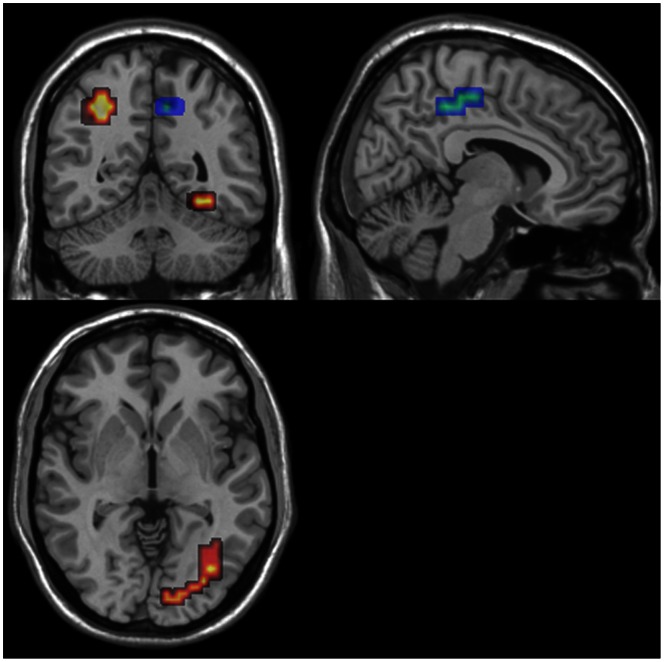
Between-group differences showing regions of greater activation on the EFT for AN (Red; p<0.009, FDR corrected) and for HC (Blue; p<0.01, FDR corrected).

The analyses revealed no group × task interaction. A main of effect of group was found when task conditions (SEF and CEF) were collapsed, in which there was greater activation in the precuneus in HC and greater activation in the fusiform gyrus in AN (Table 3, Figure 2). There was a trend for greater activation in the superior parietal lobule in AN.

**Table 3:** Cluster properties showing a difference between groups on the EFT (SEF and CEF collapsed).

Region	Size	Mass	TalairachCoordinates			Direction	p
			X	Y	Z		
Precuneus	76	0.29	10.8	−44.4	36.9	HC>AN	.004
R FusiformGyrus	56	0.43	36.1	−56.3	−18.2	AN>HC	.003
L SuperiorParietal Lobule	39	0.18	−25.3	−59.2	42.4	AN>HC	.009^a^

a This cluster did not survive correction for multiple comparisons.

## Discussion

The aim of this study was to explore the neural correlates of enhanced attention to local detail in AN using the EFT. To the best of our knowledge this is the first fMRI study of enhanced attention to local detail in AN. Unlike previous behavioural investigations, we did not find faster reaction times or greater accuracy in AN on the EFT [18], [25] and the AN group actually made more errors on the EFT than the HC group for both the simple and complex figures. However, the aforementioned studies used trial durations of up to 60 seconds, where the current study limited the time to 10 seconds per trial. Behavioural studies in ASD using longer durations of up to 120 seconds per trial find greater accuracy and faster reaction time [46]–[48], but at shorter durations in fMRI studies there is no difference in performance compared to HC [26], [27], [29], [30]. Thus differences in trial duration could contribute to the difference in findings. With regards to the paradigm itself, the current study required participants to choose between two simultaneously presented figures where the target geometrical shape is always present. Previous studies required participants to indicate whether the shape was present or not when only a single figure was presented. This could mean that a different strategy is being used to perform the task.

In line with their performance, we did not find a group × task interaction and instead found a main effect of group. The HC group showed greater activation in the precuneus, a brain region known to be implicated in visuospatial imagery and more specifically, in shifting attention between targets [49]. This would suggest that HC are more engaged in the visual search component of the task. This is in accordance with previous findings from Ring et al [26] who found greater activation in the precuneus in HC using a comparable presentation time of 15 seconds with the same type of figures. However, Damarla et al [27] found greater activation in the precuneus in people with ASD using a similar low-level baseline and a presentation time of 12 seconds, although these authors constructed a different type of complex figures (comprised of square blocks forming a three dimensional shape). The AN group showed greater activation in the right fusiform gyrus (extending into the cerebellum), a region that has been associated with object perception rather than low-level features of visual stimuli [50] and, more recently, has been suggested to be involved in the recognition of objects through simultaneous spatial integration of features [51], [52]. The lateralization of this activation is in accordance with lateralized object recognition models, suggesting that the right hemisphere focuses more on conjoining features as opposed to isolating features [51], [53]. These findings are in line with Ring et al [26], who reported greater activation in ventral occipital regions in ASD and greater activation in dorsal occipital-parietal regions in HC on the EFT. These findings would suggest that similar to ASD, AN use an alternative cognitive strategy to HC to perform the task.

Similar to ASD studies using fMRI, we find no differences in reaction time but clear differences in neural activation [26], [27], [29], [30]. In contrast to these studies, we find that HC are better than AN at discriminating between two complex figures to discern which one contains a simple geometrical shape. This was demonstrated by the clear ceiling effect of 100% accuracy on the simple figures trials and is in accordance with the notion of different cognitive strategies on the task as a whole (e.g. HC demonstrate greater visuospatial searching and AN employ a more object recognition-based approach). However, it is important to note that the current paradigm has not been used in ASD studies, therefore no direct comparison can be made.

Considering the recent criticism of the EFT, its validity as a measure of weak central coherence or enhanced attention to local detail needs to be further addressed [54]. However, behavioural studies in AN using various different tasks consistently report weak central coherence [5]. Studies employing the Rey-Osterrieth Complex Figure Test (RCFT) have found greater accuracy but a poorer performance (style and central coherence index) due to participants employing a local strategy of copying the figure with greater attention to details [18], [20], [25], [55]. On the Matching Familiar Figures Test (MFFT) [56] people with AN have shown greater accuracy and faster response times, illustrating greater detail-focused processing [19]. Finally, people with AN show poorer performance on the Fragmented Picture Task (FPT), which requires global integration of constituent parts. The nature of these latter two tasks (MFF and FPT) makes it a more suitable choice for future fMRI studies assessing enhanced attention to local detail and central coherence.

Despite not replicating previous behavioural findings, the size of the cohort and the stringent statistical methods employed for fMRI analysis strengthen the argument of different strategies employed in AN and HC. While brain structure alterations have been reported in AN [57], it is unlikely to account for the findings presented in this study. If the changes in neural activation were due to structural abnormalities, one would expect to find greater activation solely in HC in these regions. This finding suggests that central coherence should remain an important factor to address in cognitive therapies. In particular, cognitive remediation therapy (CRT) aims to improve everyday functioning through improvement of neurocognitive abilities in domains such as central coherence by introducing and facilitating “bigger picture” thinking strategies [58], [59]. Future longitudinal studies should aim to address not only the cognitive style underlying enhanced attention to local detail, but also explore the possible cognitive and neural changes following therapies that focus on appropriate adjustment of cognitive strategies. Additionally, recruitment of AN samples should encompass screening and exclusion of those with ASD in order to ensure that results do not merely reflect the influence of autistic traits but more comprehensively portray the pathology of AN.

## Supporting Information

Table S1 **Behavioural studies exploring Central Coherence in AN.**
(DOCX)

Table S2 **fMRI findings in ASD using the EFT.**
(DOCX)
