# Supplementary material for: Evaluation of Enhanced Attention to Local Detail in Anorexia Nervosa Using the Embedded Figures Test; an fMRI Study
Source: PLoS One. 2013 May 14;8(5):e63964. doi: 10.1371/journal.pone.0063964 (PMC3653828; doi:10.1371/journal.pone.0063964)
Supplement: Table S1 — Behavioural studies exploring Central Coherence in AN. (DOCX) [file pone.0063964.s001.docx]

**Supplementary Table 1.** Behavioural studies exploring Central Coherence in AN

| Authors | Sample | Task (measures) | Findings | Comments |
| --- | --- | --- | --- | --- |
| Gillberg et al 2007 | 47 AN  51 HC | WAIS-R Object Assembly subtest | AN < HC subscore |  |
| Southgate et al 2008 | 20 AN, 14 BN  26 HC | Matching Familiar Figures Test (visual search paradigm; ‘reflection–impulsivity’ and a bias  towards detail level processing, ‘weak central coherence’) | Greater efficiency (performance style) AN  i.e. superior accuracy and faster response times |  |
| Lopez et al 2008 | 42 AN  42 HC | Embedded Figures Test  Rey-Osterieth Complex Figure Test (RCFT)  Sentence Completion Test | AN > HC on EFT  Poorer performance on RCFT, but with better accuracy  Long hesitations SCT | Women with AN have  strengths in tasks requiring local processing (EFT) and weaknesses on tasks benefited by global processing (RCFT & SCT). |
| Tenconi et al 2010 | 153 AN (60 AN, 63 AN-WR, 30 complete recovered AN)  28 unaffected siblings  120 HC | RCFT (Central Coherence Index)  WAIS-R Object Assembly subtest  Overlapping Figures Test | AN showed poor performance in accuracy on RCFT, low coherence index, Object Assembly test & OFT  Performance of healthy siblings is in an intermediate position.  AN & siblings differed significantly on coherence index | All the tests investigating central coherence provided support for the presence of weak central coherence in AN |
| Roberts et al 2011 | 128 ED (35 AN-R, 33 AN-BP, 30 BN, 30 AN-WR)  50 unaffected siblings of ED  88 HC | RCFT  GEFT | AN faster on GEFT than HC  AN had lower coherence index on RCFT  AN-WR were faster on GEFT than HC  No difference siblings AN vs. AN  Trend towards siblings AN faster GEFT vs. controls  Lower coherence index siblings AN vs. HC |  |
| Oldershaw et al 2011 | 40 AN (adults)  31 ASD (adolescents) | Group Embedded Figure Test (for AN)  EFT (for ASD) | AN = ASD | Both used the 60s duration |
| Danner et al 2012 | 16 AN (10 AN-R & 6 AN-BP)  15 AN-WR (11 AN-R & 4 AN-BP)  15 HC | Rey–Osterreith Complex Figure Test (RCFT) | No group differences were found for the RCFT. | In this task, participants were asked to copy and recall (after an interval of 3 min) a complex figure. |
| Lindner et al 2012 | 100 AN-WR  100 HC | RCFT | Greater accuracy in AN vs. HC  A trend towards a local strategy in AN compared to a global strategy in HC |  |
| Allen et al 2012 | 58 ED  592 HC  (adolescents) | Groton Maze Learning Task (Executive  functioning,  including set-shifting  and global processing) | ED group made more legal, rulebreak,and perseverative errors  ED showed less efficient performance | GMLT requires participants to find and move through a  28-step pathway hidden  within a 10x10 grid  (maze), using a  predefined set of rules  and responding to  corrective feedback |
| Stedal et al 2012 | 155 AN (114 adults & 41 adolescent/children; >18 years)  Age range 9-27 | RCFT (Central Coherence Index) | Lack of coherence in the drawing process of the complex figure | Patients are somewhat more inclined to begin the drawing process by focusing on the detailed features of the figure |
| Harrison et al 2012 | 100 ED (50 AN & 50 BN)  35 AN recovered  90 HC | Fragmented Pictures Test (FPT)  RCFT  GEFT | Large to medium effect sizes are reported for all three tasks with the exception that small effect sizes were reported on the FPT in BN and AN recovered | Principal components analysis was used instead of comparing groups on behavioural measures |
| Kanakam et al 2012 | Total of 114 female participants:  26 MZ and 10 DZ twin pairs where at least one had been diagnosed with an ED (53 probands & 19 non-ED co-twins)  17 MZ and 4 DZ control twins | RCFT  GEFT | Monozygotic twins were more similar in performance than dizygotic twins in both tasks  No difference in ED co-twins vs. HC co- twins on RCFT or GEFT | Higher central coherence index in probands who were underweight compared to weight-recovered probands  Stronger local processing was found in AN probands on GEFT  For the RCFT, non-ED co-twins had lower scores than controls with medium effect size |
