# Supplementary material for: Evaluation of Enhanced Attention to Local Detail in Anorexia Nervosa Using the Embedded Figures Test; an fMRI Study
Source: PLoS One. 2013 May 14;8(5):e63964. doi: 10.1371/journal.pone.0063964 (PMC3653828; doi:10.1371/journal.pone.0063964)
Supplement: Table S2 — fMRI findings in ASD using the EFT. (DOCX) [file pone.0063964.s002.docx]

**Table S2:** fMRI findings in ASD using the EFT

| Author | Sample | Task | Contrast | Performance | Comment |
| --- | --- | --- | --- | --- | --- |
| Ring et al 1999 | 6 ASD (m/f: 4/2)  12 HC (6/6) | Standard EFT (FORM A);  12 shapes  8 target figures  ABA design  Baseline: blank screen  15s presentation  2 trials per block | **ASD>HC:**  R Cuneus  R Inferior Occipital Gyrus  R Middle Occipital Gyrus  **HC>ASD:**  R Superior Parietal Lobule  L Precuneus  L Middle Occipital Gyrus  Cuneus (bilateral)  R Inferior Frontal Gyrus  R Middle Frontal Gyrus  L Superior Occipital Gyrus  R Supramarginal Gyrus | No significant difference | Only 1 shape with figure next to it |
| Baron-Cohen et al 2006 | 6 Mothers of a child with ASD  6 Fathers of a child with ASD  6 Healthy females  6 Healthy males | See Ring et al 1999 | **HC > P_ASD:**  R Middle Occipital Gyrus  L Lingual Gyrus  **F > M:**  R Middle Occipital Gyrus  L Lingual Gyrus (Not Pooled)  L Inferior Occipital Gyrus (Pooled)  **Group*Sex Interaction:**  L Middle Occipital Gyrus  **F > Mothers:**  R Middle Occipital Gyrus  L Lingual Gyrus  **M>Mothers:**  L Inferior Occipital Gyrus  **M>Fathers:**  R Middle Occipital Gyrus  L Fusiform Gyrus  **F > M > Mothers=Fathers:**  L Cuneus  R middle occipital gyrus | No significant difference | Difference in age between parents and controls |
| Manjaly et al 2007 | 12 ASD (9 asperger & 3 HFA)  12 HC | 3 tasks:  **EFT:**  12 figures  50% of trials did have target shape  **CT:**  Matching shape with outline in figure  **Baseline:**  Triangle vs. square  3s presentation per trial in a block | **ASD & HC (EFT>baseline AND CT>baseline):**  Middle Occipital Gyrus  Intraparietal Sulcus  Fusiform Gyrus  Inferior Occipital Gyrus  Lingual Gyrus  Thalamus  Superior Parietal Gyrus  Anterior Insula  **ASD>HC:**  none | Task*group interaction  (ASD slower on control task) | Control task was similar to the EFT but was a matching instead of a searching task |
| Lee et al 2007 | 17 ASD (8 HFA & 9 Asperger)  14 HC  (children) | Self-paced trials within blocks of 30s | Trend towards reduced activation in ASD in superior parietal lobule and occipital lobe | No significant difference |  |
| Damarla et al 2010 | 13 HFA  13 matched HC | **12 test items, 8 matching shapes**  **12s presentation**  **Only 1 shape**  **with figure next to it**  **Baseline was a fixation cross**  **4 out of 12 were not matching** | **ASD>HC:**  L Postcentral, Superior Parietal & Inferior Parietal  R Superior Parietal, Precuneus & Inferior Parietal  R Postcentral, Superior Parietal & Inferior Parietal  L Superior Parietal, Precuneus & Inferior Parietal  R Superior Occipital, Middle Occipital & Cuneus  **HC > ASD:**  L Superior Frontal & Superior Medial Frontal  L Middle Frontal & Precentral  L Thalamus & Putamen  L Supramarginal & Inferior Parietal  L Inferior Parietal & Angular | No significant difference | Paradigm consisted of EFT & fixation cross  Lower frontal-posterior functional connectivity |
| Spencer et al 2012a | 40 ASD (classic or Asperger) (m/f: 34:4)  40 Unaffected siblings (12:28)  40 HC (20:20)  adolescents | See Manjaly et al 2007 (without baseline)  8 blocks EFT  8 blocks CT  (Six 4s stimuli per block)  EFT vs. Control Task (EFT>CT) | **ASD>HC:**  L Middle Temporal Gyrus  L Inferior Frontal Gyrus  R Superior Temporal Sulcus  R Inferior Temporal Sulcus  L Angular Gyrus  **HC>ASD:**  L Fusiform Gyrus  L V3  L Supramarginal Gyrus  L Pre-Motor Cortex  **Siblings>HC:**  L Primary Motor Cortex  L Anterior Superior Temporal Sulcus  L Inferior Frontal Gyrus  Insula (Bilateral) |  | Opposite of earlier findings |
| Spencer et al 2012b | 40 ASD (classic or Asperger)  40 Unaffected siblings (12:28)  40 HC (20:20)  adolescents | EFT vs. Control Task (CT>EFT)  Task-related deactivation | **HC>ASD:**  R Inferior Parietal Cortex  Posterior Cingulate Cortex  L Inferior Parietal Cortex  **HC>Siblings:**  R Inferior Parietal Cortex  Posterior Cingulate Cortex | No significant difference | Contrasts indicate greater deactivation instead of greater activation |
